# Detection of antibodies in suspected autoimmune encephalitis diseases using machine learning

**DOI:** 10.1038/s41598-025-95815-z

**Published:** 2025-03-31

**Authors:** Manfred Musigmann, Christine Spiekers, Jacob Stake, Burak Han Akkurt, Nabila Gala Nacul Mora, Thomas Sartoretti, Walter Heindel, Manoj Mannil

**Affiliations:** 1 University Clinic for Radiology, University of Münster and University Hospital Münster, Albert-Schweitzer-Campus 1, 48149 Münster, Germany; 2 Diagnostic and Interventional Radiology, University Hospital Zurich, University of Zurich, Zurich, Switzerland

**Keywords:** Autoimmune encephalitis, Seropositivity, Hippocampus, Machine learning, Radiomics, MRI, Neuroimaging, Biomarkers, Diseases, Medical research, Neurology, Oncology

## Abstract

In our study, we aim to predict the antibody serostatus of patients with suspected autoimmune encephalitis (AE) using machine learning based on pre-contrast T2-weighted MR images acquired at symptom onset. A confirmation of seropositivity is of great importance for a reliable diagnosis in suspected AE cases. The cohort used in our study comprises 98 patients diagnosed with AE. 57 of these patients had previously tested positive for autoantibodies associated with AE. In contrast, no antibodies were detected in the remaining 41 patients. A manual bilateral segmentation of the hippocampus was performed using the open-source software 3D Slicer on T2-weighted MR-images. Subsequently, 107 Radiomics features were extracted from each T2-weighted MR image utilizing the open source PyRadiomics software package. Our study cohort was randomly divided into training and independent test data. Five conventional machine learning algorithms and a neural network were tested regarding their ability to differentiate between seropositive and seronegative patients. All performance values were determined based on independent test data. Our final model includes six features and is based on a Lasso regression. Using independent test data, this model yields a mean AUC of 0.950, a mean accuracy of 0.892, a mean sensitivity of 0.892 and a mean specificity of 0.891 in predicting antibody serostatus in patients with suspected AE. Our results show that Radiomics-based machine learning is a very promising method for predicting serostatus of suspected AE patients and can thus help to confirm the diagnosis. In the future, such methods could facilitate and accelerate the diagnosis of AE even before the results of specific laboratory tests are available, allowing patients to benefit more quickly from a reliable treatment strategy.

## Introduction

Autoimmune encephalitis (AE) is a term for heterogeneous conditions in which the body’s immune system attacks the brain, causing inflammation^1^. Characteristically, inflammation is present without infection as a result of a misdirected immune response against self-antigens of the central nervous system. Believed to be extremely rare in the past, recent studies have concluded that the prevalence of AE may be almost as high as that of infectious encephalitis^2,3^. Due to the variety of nonspecific symptoms, some authors argue that AE is still underdiagnosed^4^. Symptoms include altered mental abilities, short-term memory disorders, and seizures^5,6^. The treatment of AE is currently based on the administration of glucocorticoids, immunoglobulins, plasmapheresis, and various psychiatric medications, the efficacy of which impacts the prognosis^7^. Early detection and diagnosis of AE can expedite the optimal administration of immunotherapy and thus significantly improve prognosis^8^. As early treatment has been shown to improve outcome, rapid diagnosis is of great importance^9,10^.

In about half of all cases, patients with AE exhibit detectable antibodies against structures of the central nervous system^11,12^. These antibodies can be directed either against intracellular proteins or extracellular antigens^13^. The first group, antibodies targeting intracellular antigens – such as anti-Hu or anti-Ma – often occur in conjunction with an underlying neoplasm and usually respond poorly to first-line immunotherapy^13^. Corresponding antibodies occur simultaneously with a cytotoxic T-cell reaction causing neuronal damage^14–16^. In the second group – comprised of antibodies directed against extracellular antigens such as anti-NMDAR, anti-LGI1, and anti-AMPAR^17,18^ – neuronal function is suppressed in a titer-dependent manner^19^. This effect is often reversible under immunotherapy, which frequently results in a better prognosis^20–22^. Finally, in the third group, the seronegative cases, it remains uncertain whether the antibodies cannot be detected with current test methods or are entirely absent. In these approximately 50% of all cases^23^ of suspected AE, clinical criteria are used for the diagnosis^6^.

The expertise required for diagnosis limits the ability of inexperienced clinicians to diagnose the condition^6^. As previously explained, numerous antibodies can point to the diagnosis, but magnetic resonance imaging (MRI) is particularly supportive^24^. Studies show frequent involvement of the limbic system, especially in the hippocampus^25^, which is also highlighted in histopathological studies^26^. Graus et al. defined specific criteria for diagnosis, including subacute onset, abnormalities in the T2-weighted MRI scan, EEG abnormalities, and CSF pleocytosis, with no other diagnosis fulfilling these criteria^6^. MRI imaging at symptom onset is of great importance for diagnosis, but image morphological correlates are not always unambiguous. They can range from unremarkable imaging findings to hippocampal atrophy, complicating the diagnosis^27^. In the limbic system, characteristic MRI findings are most frequently seen in T2-weighted images, including hyperintense structures or oedematous enlargement to atrophy in advanced disease stages^28^.

Due to the problems described regarding a reliable and rapid diagnosis of AE, new and improved analytical methods are of great interest. The use of Radiomics is such a new and promising approach. Radiomics characterizes morphological image anomalies using quantitative information extracted from radiological images^29^. The analysis is based on the examination of signal intensity gradations at a voxel level that are not perceptible to the human eye. In this way, Radiomics may detect disease patterns not recognized by conventional techniques^30–32^. Moreover, Radiomics complements conventional MRI imaging and converts specific MR image components into medical data to explain causal pathophysiology^33^. Common Radiomics software products usually calculate many different features, in correspondingly high-dimensional data sets that are often difficult to analyze using conventional statistical approaches^34^. Artificial intelligence (AI) methods have become an indispensable tool for corresponding analyses and represent an emerging area of current medical research^35^. Machine learning (ML) algorithms in turn represent a subgroup of AI. Supervised machine learning algorithms are first trained with known (labeled) data sets to enable them to make appropriate decisions/diagnoses with unknown data sets subsequently. ML is expected to help detect diseases earlier and facilitate diagnosis, thereby improving patient outcomes^33^. Machine learning has already made significant strides in medical research, particularly in diagnosing brain tumors^36^. Among other findings, Radiomics based on MRI images in combination with machine learning was able to predict IDH and ATRX mutation status in glioma patients with high accuracy^37–39^. The non-invasive determination of these molecular markers is of high importance according to the WHO classification 2021 for diffuse gliomas of the adult type^40^.

Following these ideas, we investigate in our current study the feasibility of a non-invasive determination of seropositivity in patients with suspected AE. Specifically, we analyze whether we can predict the serostatus of patients with suspected AE using machine learning based on pre-contrast T2-weighted MRI images acquired at symptom onset. The radiomic features used are based on a bilateral segmentation of the hippocampus. As edematous enlargement of the hippocampus is frequently observed in AE, we expected shape-based features to be of high importance for our analysis. Five conventional machine learning algorithms and a neural network are tested regarding their ability to predict serostatus (i.e. to distinguish between detectable and no detectable antibodies). Such machine learning-based methods could potentially simplify and accelerate the complex diagnostic process for patients with suspected AE in the future. In seronegative patients, on the other hand, laboratory capacities could be freed up by avoiding unnecessary antibody testing in these cases. Overall, the time between initial presentation an initiation of therapy could be shortened, which – as emphasized – could improve the prognosis of patients.

## Materials and methods

**Fig. 1:**
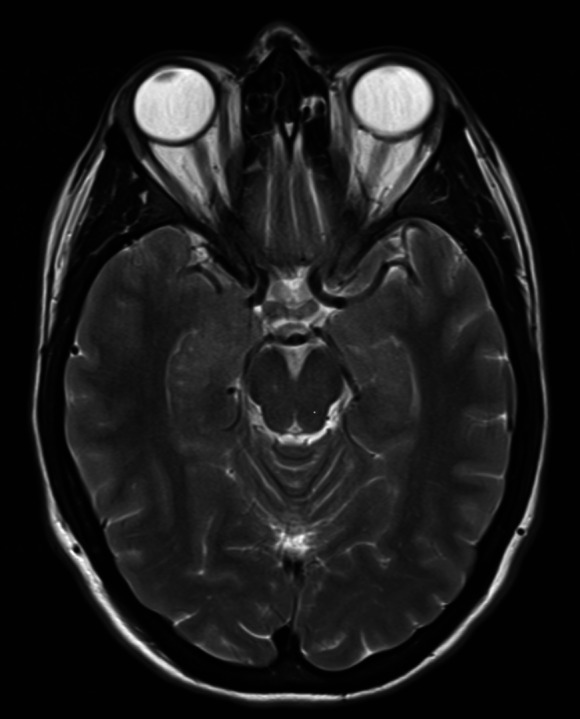
Pre-contrasted T2-weighted MR image (acquired at symptom onset) from a 31-year-old seropositive woman who presented with altered mental status and seizures. Enlarged oedematous hippocampus.

Our study was performed in compliance with the Declaration of Helsinki^41^ and approved by the local ethics committee (Ärztekammer Westfalen-Lippe (ÄKWL) Münster 2022-298-f-S). Due to its retrospective nature, written informed consent was waived (Ärztekammer Westfalen Lippe and University of Münster). Our aim is to determine the antibody status in patients with suspected AE using machine learning based on magnetic resonance images. We distinguish between the two possible outcomes of previously detected (i.e. seropositive patients) and undetected AE-specific autoantibodies (i.e. seronegative patients). Our hospital’s database was retrospectively searched for patients diagnosed with AE between March 2009 and February 2022. For this purpose, the patient records were screened and evaluated according to the diagnosis and antibody status listed in the respective discharge letters. Our analyses are based on pre-contrast T2-weighted MR images acquired at symptom onset. As an example, Fig. 1 shows a corresponding T2-weighted MR image of a 31-year-old seropositive woman. All MR images were acquired using 1.5T and 3T scanners at our institution and secondary care centers.

The bilateral segmentation of the region of interest (ROI), i.e. of the hippocampus, was performed in the axial direction. Initially, we included 109 patients in our study. We excluded MRI images with significant artifacts that could affect the extraction of radiomic features. In addition, MRI images from external centers with non-standardized MRI acquisition protocols (source variability) were also excluded. All images were manually inspected to ensure sufficient quality for hippocampus segmentation. In total, the criteria mentioned resulted in the exclusion of 11 patients. Apart from these 11 patients, no other patients were excluded. Antibodies were detected in 57 of the remaining 98 patients and no antibodies were detected in 41 patients. The most common antibodies detected were anti-GAD65- (18 patients), anti-LGI1- (10 patients) and anti-CASPR2-AB (7 patients). The demographic characteristics of the final study cohort used for the machine-learning based determination of antibody status (detected/undetected antibodies) are summarized in Table 1.

**Table 1:** Demographic characteristics of the patients in our autoimmune encephalitis study cohort for predicting antibody status using machine learning.

	Training data	Independent test data	Total data
Number of patients	79	19	98
Antibodies detected (in %)			
Yes	58.23	57.89	58.16
No	41.77	42.11	41.84
Gender (in %)			
Female with antibodies	27.66	27.10	27.55
Female without antibodies	19.05	20.79	19.39
Male with antibodies	30.57	30.79	30.61
Male without antibodies	22.72	21.32	22.45
Mean age (in years)			
Mean age: patients with antibodies	55.53	55.42	55.51
Mean age: patients without antibodies	45.58	45.85	45.63

### Radiomics

Bilateral segmentation of the hippocampus was manually performed using the 3D Slicer open-source software platform (version 4.10, http://www.slicer.org) and utilizing the Segmentation Wizard plugin. Expert consensus was sought in anatomically difficult bilateral segmentations. A detailed description of the anatomical reference points used can be found in the segmentation protocol by Moore et al.^42^. To ensure the comparability of the MR images used, standardized preprocessing was applied to all images. For this purpose, all MR images were resampled to a voxel size of 2 × 2 × 2 mm² to ensure uniformity by spatial resampling. A uniform bin width of 64 was used for histogram normalization to standardize intensity ranges across images and provide noise reduction.

A total of 107 radiomic features were determined using the PyRadiomisc package. The package is available as an implementable plugin for the 3D Slicer platform. The radiomic features were extracted by hand-delineated regions of interest (ROI) from the MR images of each patient. These 107 features are composed of 18 first order statistics features, 14 shape-based features, 24 Gy level co-occurrence matrix features, 16 Gy level run length matrix features, 16 Gy level size zone matrix features, 5 neighbouring gray tone difference matrix features and 14 Gy level dependence matrix features. In addition, our database contained the age of the patients at the time of the MRI scan and their gender. All features were z-score transformed and subjected to a 95% correlation filter to exclude redundant information.

### Statistical analysis

Statistical analysis was performed using R software (version 4.1.2). The 98 patients in our final study cohort were randomly assigned to a training group and an independent test group. We used a stratified 4:1 ratio with a balanced distribution of patients with detected and undetected antibodies between these two groups (see Table 1), i.e. with seropositive and seronegative patients. The training data (comprising 80% of the total data) were used for feature preselection and subsequent model development. Six different machine learning algorithms were tested to determine antibody status (i.e. to distinguish between the two possible outcomes of detectable and no detectable antibodies). Specifically, we used the Random forest algorithm, Naive Bayes, a linear discriminant analysis (LDA), a Lasso regression (Least absolute shrinkage and selection operator) as well as a ridge regression and a neural network. In each case, the same algorithm was used for feature preselection and subsequent model development. Hyperparameters included in the models were optimised using 10-fold cross-validation. The models themselves were optimised by maximising the area under the curve (AUC) of the receiver operator characteristic (ROC). The performance of the models was determined in each case using the remaining 20% of the total data, i.e. using completely independent test data. Strict separation of data into training data and independent test data ensures that the test data has no influence on the model training. Consequently, the performance values determined with the independent test data cannot be distorted by data leakage. Performance was determined in terms of AUC, accuracy, sensitivity, specificity, positive predictive value (PPV) and negative predictive value (NPV). The accuracy describes the proportion of correctly predicted cases overall, the sensitivity the proportion of correctly predicted cases with detected antibodies and the specificity the proportion of correctly predicted cases without detectable antibodies. The PPV, on the other hand, indicates the proportion of correctly predicted cases with detected antibodies in relation to all predicted cases with detected antibodies. The corresponding ratio for undetected antibodies is given by the NPV.

The model performance achieved depends on the number of features included in the models. Therefore, we developed all models with an increasing number of features, starting with a single-feature model in each case. In each model with a predefined number of features, the most important features were included in the models. These features were determined beforehand using the “varImp”-function in R (varImp = variable importance). This function calculates the performance gain achieved by each individual feature. In this way, the varImp-function determines the most important features in each case. We determined the optimal number of model features to include by analysing which model complexity (in terms of the number of features included) resulted in the highest model performance in relation to the independent test data. This approach minimises the risk of possible overfitting.

**Fig. 2:**
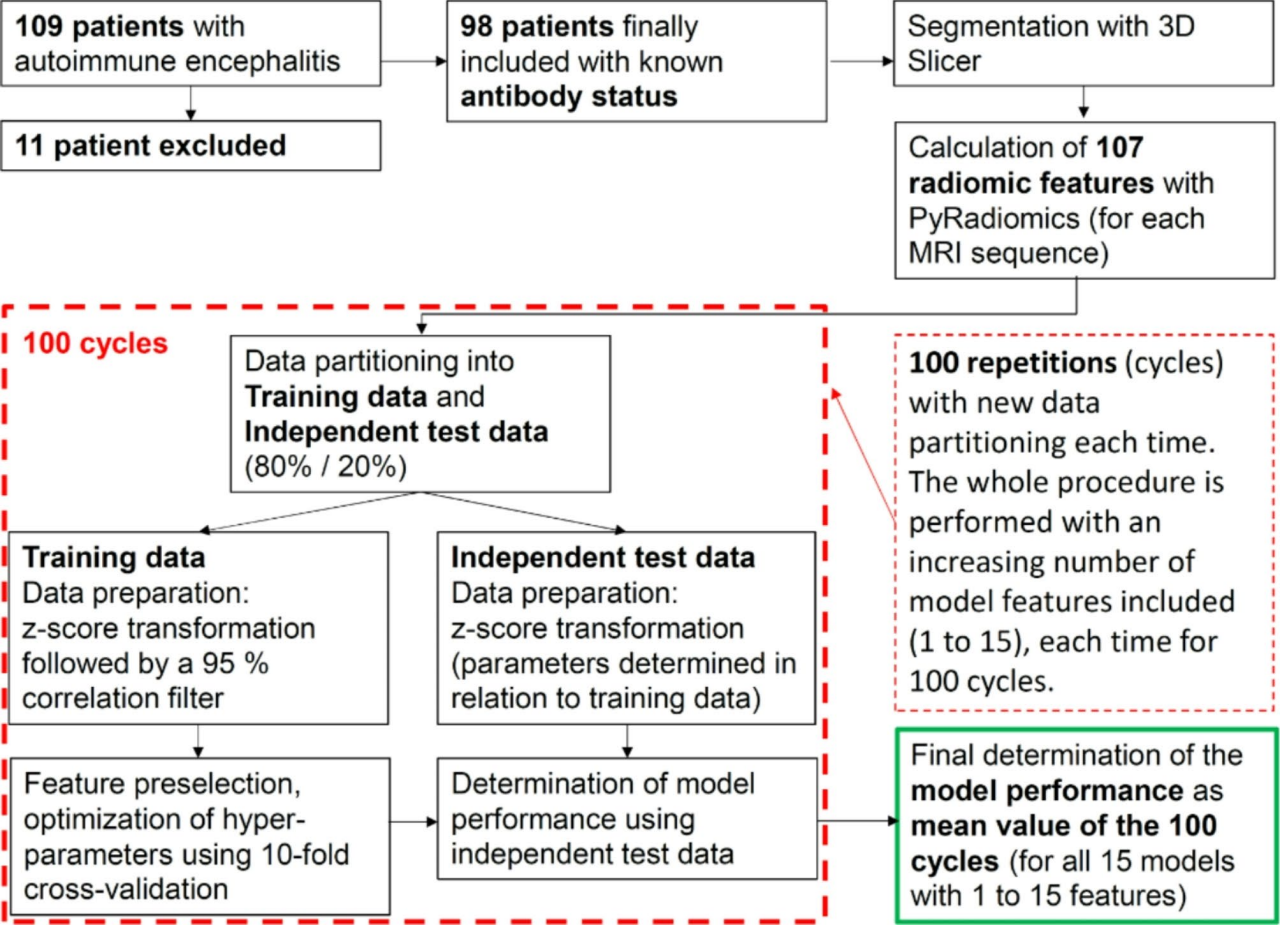
Flowchart describing the methodological approach. For each tested machine learning algorithm (i.e. Naïve Bayes, LDA, Lasso regression, ridge regression, Random forest and a neural network), a total of 15 models are developed with an increasing number (1 to 15) of model features included. Each of these models is developed 100 times, each time with a new data partitioning, and subsequently tested. The final determination of the performance of each model is calculated as the average of the 100 cycles.

Since the model performance achieved depends not only on the number of features used, but also on the division of the data into training and independent test data, we performed the data partitioning and subsequent full model development and testing 100 times for each model using 100 different data partitions. Accordingly, the values shown in Table 1 were also calculated as mean values over these 100 runs. Without exception, we calculated all performance values as mean values of these 100 runs. We also determined the 95% confidence intervals. To facilitate understanding, we have described the entire process in a flow chart (see Fig. 2). The exact procedure is described in more detail here.

## Results for predicting antibody status

**Fig. 3:**
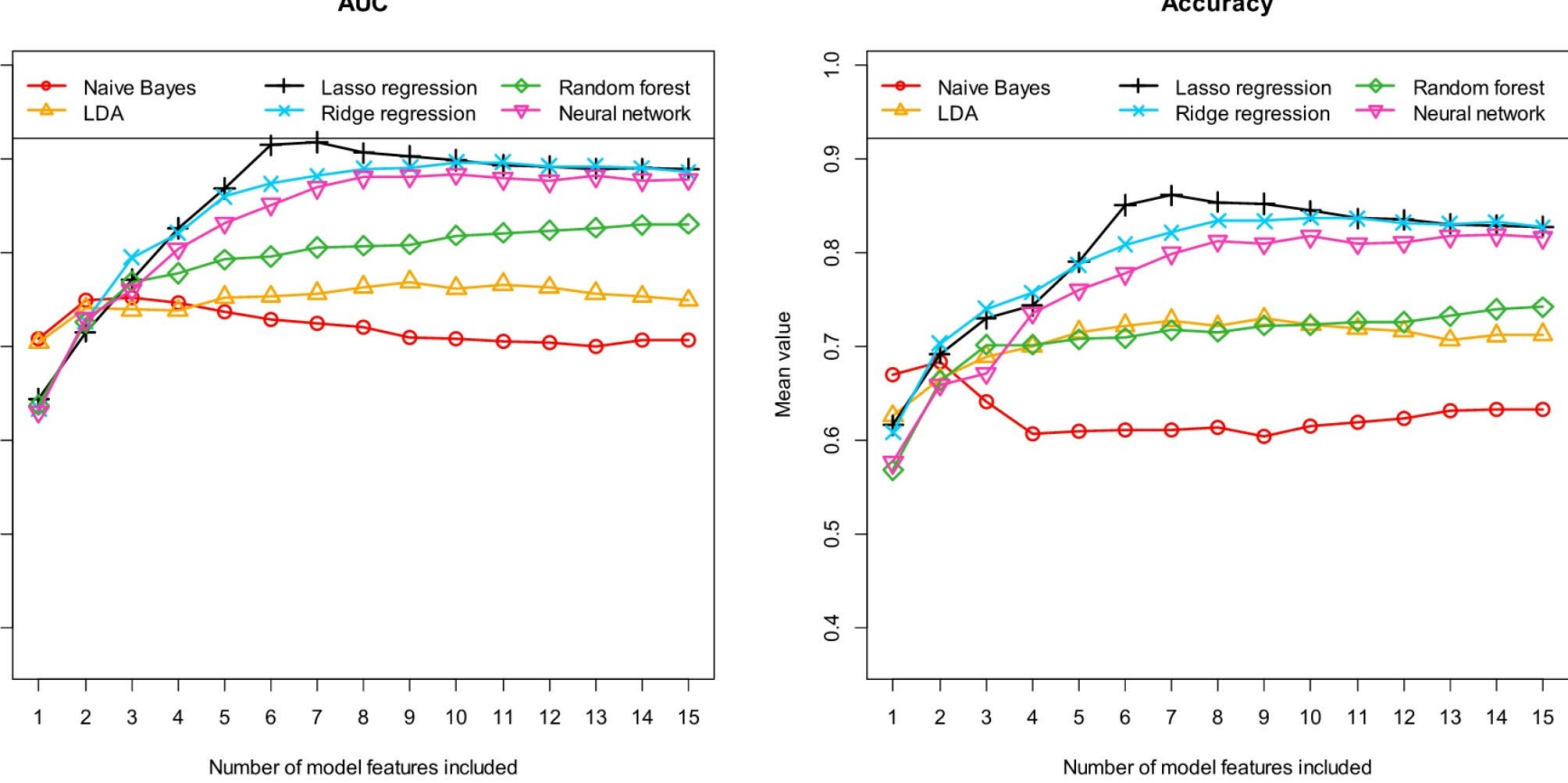
Prediction of antibody status (detectable/no detectable antibodies) in patients with suspected autoimmune encephalitis: Area under the curve (*AUC*) and *accuracy* for the independent test samples, calculated as means of 100 repetitions (100 cycles) depending on the number of model features included. Six different machine learning algorithms (as indicated in the figures) were tested for feature preselection and subsequent model construction.

**Fig. 4:**
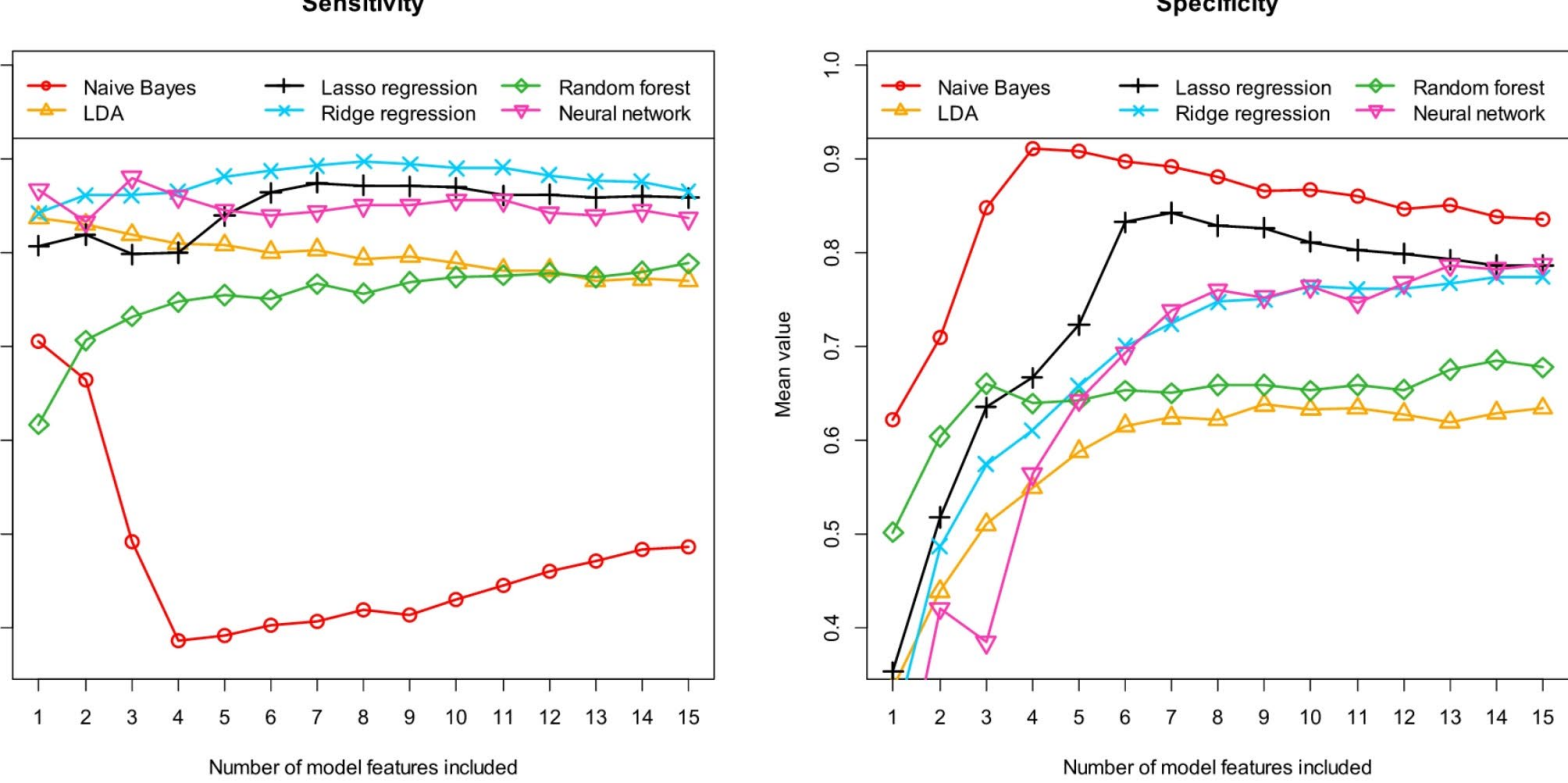
Prediction of antibody status (detectable/no detectable antibodies) in patients with suspected autoimmune encephalitis: *Sensitivity* and *specificity* for the independent test samples, calculated as means of 100 repetitions (100 cycles) depending on the number of model features included. Six different machine learning algorithms (as indicated in the figures) were tested for feature preselection and subsequent model construction.

**Fig. 5:**
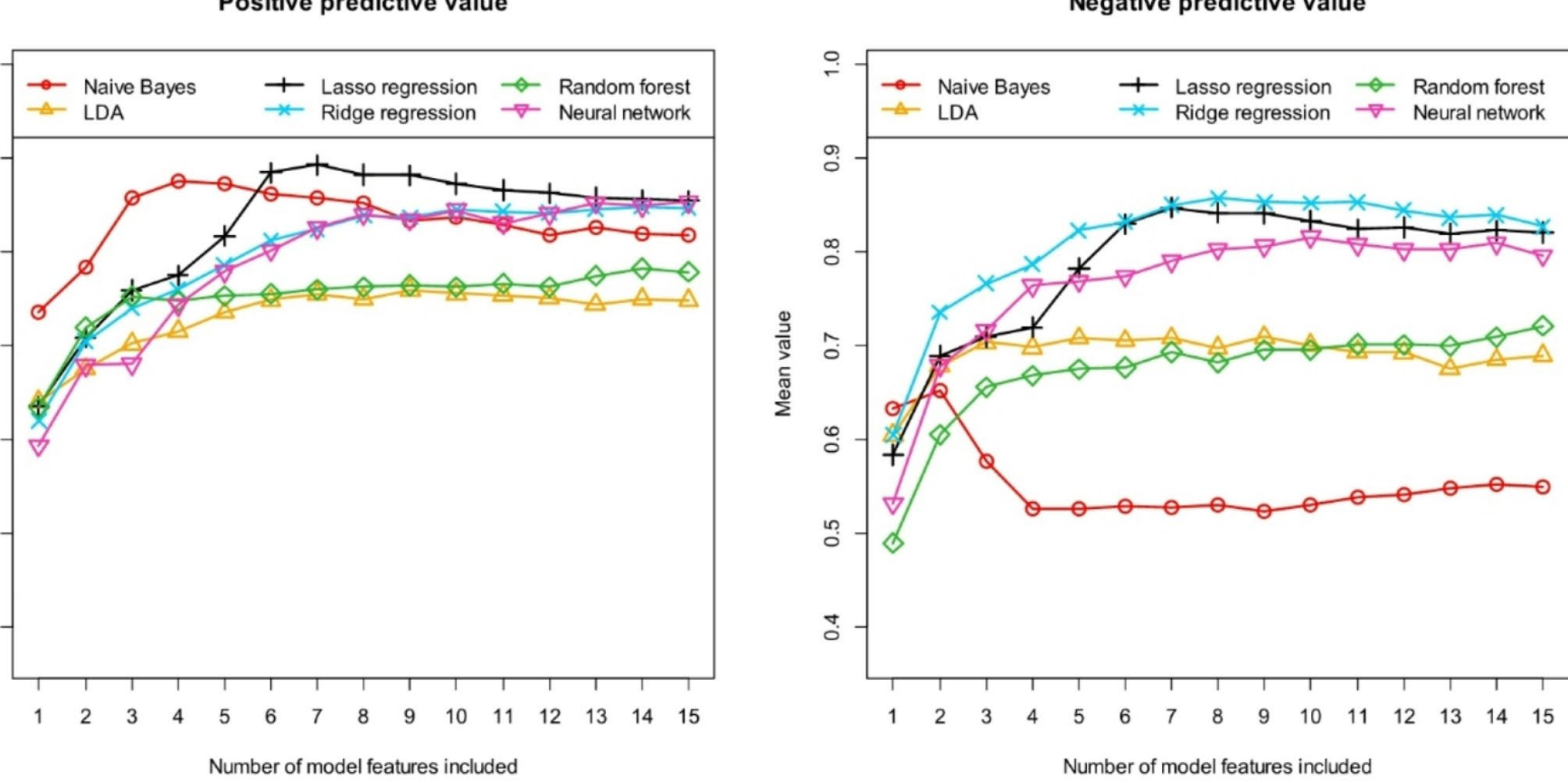
Prediction of antibody status (detectable/no detectable antibodies) in patients with suspected autoimmune encephalitis: *Positive predictive value (PPV)* and *negative predictive value (NPV)* for the independent test samples, calculated as means of 100 repetitions (100 cycles) depending on the number of model features included. Six different machine learning algorithms (as indicated in the figures) were tested for feature preselection and subsequent model construction.

As explained, we tested six different machine learning algorithms to distinguish suspected AE patients with previously detected and undetected antibodies. Figure 3 first shows the performance achieved with these six machine learning algorithms in terms of AUC and accuracy. Accordingly, Fig. 4 describes the performance in terms of sensitivity and specificity and Fig. 5 describes the performance in terms of positive and negative predictive value. All results were calculated using the independent test samples and as mean values of 100 runs. As previously described, each of these 100 runs was performed using a new data partitioning (i.e. new training data and independent test data).

We began the analysis of our model results by first examining the stability of the individual models in terms of their feature composition. As Figs. 3, 4 and 5 show, using more than seven features does not result in a further significant increase in discriminatory power for most of the tested algorithms. As described, each individual model was completely developed 100 times using different data partitions. Therefore, the 100 associated models may differ in their feature composition. For this reason, we analyzed the differences between these 100 models. For each of the six machine learning algorithms tested, we examined how often each feature was selected during the respective 100 runs, based on the associated 7-feature models. Naive Bayes and LDA resulted in exactly the same features. The same was found for Lasso regression and ridge regression. Table 2 lists the 10 most frequently selected features for each of the six machine learning algorithms. The number of times each feature was selected during the 100 runs is given in brackets after each feature name. In addition, it is indicated in the brackets whether the respective feature correlates positively (pos.) or negatively (neg.) with the antibody status. A positive correlation means that as the value of the feature increases, the probability that antibodies can be detected also increases. For example, the age of the patients was selected in 100 of 100 runs using Lasso regression/ ridge regression. Age correlates positively with antibody status, i.e. antibodies are detected more often in older patients than in younger ones.

**Table 2:** Most important features for predicting antibody status (detectable/no detectable antibodies) in patients with suspected autoimmune encephalitis using T2-weighted MR images. Calculations are based on the 7-feature models. The numbers in brackets indicate how often the corresponding feature was selected during 100 runs and whether the feature correlates positively or negatively with the antibody status (see text).

Level of importance	Naive Bayes/LDA	Random forest	Lasso/ridge	Neural network
1	DependenceNonUniformity Normalized (100 runs, neg.)	Sphericity (98 runs, pos.)	Age_at_scan (100 runs, pos.)	Age_at_scan (100 runs, pos.)
2	LargeAreaLowGrayLevel Emphasis (100 runs, pos.)	DependenceNonUniformity Normalized (95 runs, neg.)	Sphericity (100 runs, pos.)	GrayLevelNonUniformity2 (97 runs, neg.)
3	RobustMeanAbsolute Deviation (99 runs, neg.)	LargeAreaLowGrayLevel Emphasis (86 runs, pos.)	GrayLevelNonUniformity2 (99 runs, neg.)	Sphericity (95 runs, pos.)
4	Sphericity (96 runs, pos.)	SurfaceVolumeRatio (59 runs, neg.)	Correlation (99 runs, neg.)	Correlation (90 runs, neg.)
5	Busyness (82 runs, pos.)	GrayLevelNonUniformity2 (57 runs, neg.)	Coarseness (93 runs, neg.)	Coarseness (71 runs, neg.)
6	ClusterTendency (58 runs, pos.)	RobustMeanAbsolute Deviation (56 runs, neg.)	Maximum2DDiameter Column (84 runs, neg.)	Maximum2DDiameter Column (42 runs, neg.)
7	GrayLevelNonUniformity (40 runs, pos.)	LargeAreaHighGrayLevel Emphasis (52 runs, pos.)	ClusterShade (27 runs, pos.)	Skewness (36 runs, pos.)
8	SurfaceVolumeRatio (27 runs, neg.)	Age_at_scan (28 runs, pos.)	Maximum2DDiameterSlice (24 runs, neg.)	Parenoplastic (31 runs, pos.)
9	SumEntropy (27 runs, neg.)	ShortRunEmphasis (23 runs, neg.)	LargeAreaHighGrayLevel Emphasis (18 runs, pos.)	SmallAreaLowGrayLevel Emphasis (28 runs, pos.)
10	SumAverage (21 runs, neg.)	ClusterShade (15 runs, pos.)	Flatness (11 runs, pos.	SurfaceArea (22 runs, neg.)

It is evident that the six machine learning algorithms tested result in models that are stable in terms of their key features. The feature “Sphericity” is always one of the most important features, regardless of the machine learning algorithm used. This feature was selected in almost every run. In terms of the other features, the six algorithms can be roughly divided into two groups. The first group is formed by the three algorithms Naive Bayes, LDA and Random forest. In this group, the three additional features “DependenceNonUniformityNormalized”, “LargeAreaLowGrayLevelEmphasis” and “RobustMeanAbsoluteDeviation” are particularly important. The second group consists of the three remaining algorithms: Lasso regression, ridge regression and the neural network. In this group, the four additional features “Age_at_scan”, “GrayLevelNonUniformity2”, “Correlation” and “Coarseness” are also of particular importance.

Comparing the six different machine learning algorithms tested in terms of their performance in predicting antibody status, it is obvious that Naive Bayes exhibits the worst discriminatory power. This is particularly evident regarding sensitivity and NPV. When using the Naive Bayes algorithm, the discriminatory power (see in particular Fig. 3: AUC and accuracy) cannot be increased any further from the second feature onwards. While specificity increases with additional features, sensitivity decreases again (see Fig. 4), so that accuracy remains almost the same (Fig. 3). The results clearly show that the Naive Bayes algorithm is already overfitted with only three features, which leads to very unstable performance results. In conclusion, the simple Naive Bayes algorithm is only of limited use for predicting antibody status. The discriminatory power of the linear discriminant analysis (LDA) and the Random forest algorithm is also lower compared to the other algorithms. Overall, the three algorithms Naive Bayes, LDA and Random forest thus show the three lowest discrimination values of the six tested algorithms. This is interesting in that, as already mentioned, these three algorithms essentially use different features than the three remaining algorithms (see Table 2). Apart from the fact that the three remaining machine learning algorithms, Lasso regression, ridge regression and neural network, may already be better suited for predicting the antibody status due to their model architecture, the achieved model performance may also have been positively influenced by the fact that these algorithms may have been able to determine an even more suitable multivariate combination of features. Both ridge regression and the neural network already exhibit high levels of discriminatory power. However, of the six algorithms tested, the Lasso regression performed best overall. Here, the highest discriminatory power is achieved if seven features are included in the models. Lasso regression models containing more than 7 features show a decreasing discriminatory power compared to the 7-feature model. This means that the model is overfitted if more than seven features are included. On average of 100 runs, the Lasso regression models with 7 features exhibit an AUC of 0.918 [0.728, 1.000], an accuracy of 0.861 [0.684, 1.000], a sensitivity of 0.875 [0.545, 1.000], a specificity of 0.843 [0.500, 1.000], a PPV of 0.893 [0.711, 1.000] and a NPV of 0.847 [0.600, 1.000]. The numbers in brackets indicate the 95% confidence interval.

The two features “Age_at_scan” and “Sphericity” are included in all Lasso regression models with 7 features (see Table 2), and the two further features “Correlation” and “GrayLevelNonUniformity” are included in almost all models (99 of 100 runs). The further feature “Coarseness” is included in 93% of the models and the feature “Maximum2DDiameterColumn” in 84% of all models. The most important 6 of the 7 features are therefore identical for most models. Beyond these 6 most frequently selected features, the importance of the other possible additional features decreases sharply. The feature with the seventh highest importance (“ClusterShade”) was already selected in only 27 of the 100 runs. This fact is consistent with the observation that further additional features no longer significantly increase discriminatory power compared to a model with 6 features (see Figs. 3, 4 and 5).

To determine the extent to which the achieved performance of our models depends on the exact individual model composition, we have additionally developed two models containing the 6 and 7 most frequently selected features of the 100 runs in fixed form. Thus, the first of these two models contained only the features selected in at least 84% of the previously conducted runs (i.e. the features “Age_at_scan” to “Maximum2DDiameterColumn”, see Table 2). The second model additionally contained the feature “ClusterShade” as a seventh feature. These two models were again developed 100 times using 100 different data partitions. This time, however, the features contained in the models were fixed during the 100 runs. Table 3 summarizes the classification results obtained with these 6- and 7-feature models for the case of the described fixed feature composition (columns “Fixed features”). For comparison, the table shows in addition the classification results of the models with 6 and 7 features, which were obtained in the case of a variable feature composition (columns “Different features”). All results were again calculated based on independent test data and as mean values of 100 runs. The table also contains the 95% confidence intervals of the results. It is obvious that the seventh feature does not contribute significantly to the discriminatory power.

**Table 3:** Classification results for predicting antibody status (detectable/no detectable antibodies) for The *6- and 7-feature Lasso regression models*. The results in The columns labelled “fixed features” were calculated with fixed features according to Table 2. The columns labelled “different features” show The results for The case that The features were newly determined in each of The 100 runs (i.e. The features included May be different). All results are calculated using independent test data and as mean values of 100 runs. The values in brackets indicate The 95% confidence interval.

	Models with 6 features		Models with 7 features	
	Fixed features	Different features	Fixed features	Different features
AUC	0.950 [0.836 : 1.000]	0.915 [0.739 : 1.000]	0.955 [0.852 : 1.000]	0.918 [0.728 : 1.000]
Accuracy	0.892 [0.737 : 1.000]	0.851 [0.634 : 1.000]	0.901 [0.737 : 1.000]	0.861 [0.684 : 1.000]
Sensitivity	0.892 [0.593 : 1.000]	0.864 [0.593 : 1.000]	0.898 [0.593 : 1.000]	0.875 [0.545 : 1.000]
Specificity	0.891 [0.625 : 1.000]	0.833 [0.500 : 1.000]	0.904 [0.625 : 1.000]	0.843 [0.500 : 1.000]
PPV	0.926 [0.769 : 1.000]	0.884 [0.680 : 1.000]	0.935 [0.769 : 1.000]	0.893 [0.711 : 1.000]
NPV	0.872 [0.642 : 1.000]	0.830 [0.553 : 1.000]	0.880 [0.642 : 1.000]	0.847 [0.600 : 1.000]

Our methodology with 100 repetitions is well suited to analyse the stability of a particular model approach and to compare different approaches. Regarding the question analysed in this study, the approach we chose provided very stable results. The models developed with the different features exhibit very similar discriminatory power to the models developed subsequently with fixed features. In detail, the two approaches (fixed features/different features) using 6-features resulted in the following mean performance values: mean AUC = 95.0%/91.5%, mean accuracy = 89.2%/85.1%, mean sensitivity = 89.2%/86.4%, mean specificity = 89.1%/83.3%, mean PPV = 92.6%/88.4% and mean NPV = 87.2%/83.0%. Thus, our models show very high performance in predicting antibody status in our cohort of suspected AE cases.

Since patient age proved to be such an important feature, we were also interested in determining the influence of patient age and radiomic features separately on the discriminatory power of the models. For this purpose, we developed a univariate model that included only the age of the patients and no other features. This model yielded an average AUC of 0.649 [0.421:0.863] based on the independent test data. The additional consideration of the patient’s gender (model with age + gender) did not increase the discriminatory power compared to the univariate model. Conversely, the model that included only the six most important radiomic features 2 to 7 from Table 2, column “Lasso/Ridge” (excluding patient age) yielded the following average performance values: mean AUC = 0.856 [0.683:1.000], mean accuracy = 0.802 [0.632:0.972], mean sensitivity = 0.842 [0.545:1.000], mean specificity = 0.748 [0.441:1.000], mean PPV = 0.827 [0.666:1.000], and mean NPV = 0.793 [0.545:1.000]. Random models have an AUC of 0.5, while perfect models have an AUC of 1. Thus, patient age alone (univariate) achieves 29.9% of the theoretically possible increase in discriminatory power in terms of AUC. The additional consideration of the six most important radiomic features increases the possible AUC-related gain in discriminatory power by 61.2% to a total of 91.1%. Conversely, the six most important radiomic features alone (without taking patient age into account) achieve 71.3% of the theoretically possible gain in AUC. Overall, these results clearly show that the radiomic features alone have significant discriminatory power.

Finally, we were also interested in determining the advantage of using machine learning algorithms compared to traditional algorithms such as logistic regression. Using stepwise logistic regression, we obtained (after seven steps) a mean AUC of 0.878 [0.595:1.000], a mean accuracy of 0.829 [0.579:0.972], a mean sensitivity of 0.850 [0.545:1.000], a mean specificity of 0.800 [0.441:1.000], a mean PPV of 0.860 [0.640:1.000], and mean NPV of 0.809 [0.500:1.000]. Compared to the results obtained with Lasso regression (Table 3), these performance values are similar but slightly lower. Logistic regression and Lasso regression are two related algorithms, which certainly explains the relative proximity of the results obtained. The additional regularization term included in the Lasso regression leads to the slightly higher discriminatory power when comparing these two algorithms.

## Discussion

In this proof-of-concept study, we developed an approach to predict serostatus in suspected AE using Radiomics-based machine learning algorithms. As Piao et al. have shown, early diagnosis of AE could reduce disease severity^43^. Therefore, a rapid and reliable diagnosis is of great importance. The current standard for diagnosis of AE relies on clinical symptoms, antibody status, and imaging, as described by Graus et al.^6^.

Development of new diagnostic approaches could allow for non-invasively predicting serostatus patients with suspected AE. As shown in our study, a potential new approach could be Radiomics-based machine learning. Radiomics, an emerging field of medical imaging, utilizes a large number of quantitative features extracted from radiological images. By this approach, information and disease characteristics that may be invisible to the human eye can be detected. In combination with machine learning, Radiomics enables completely new diagnostic approaches in medicine. In this way, even very complex patterns can be recognized on radiological images that may not be perceived in a human analysis. As we have shown in our study, the serostatus of patients with suspected AE can be predicted non-invasively with high accuracy using machine learning. Our analyses are based on T2-weighted MRI images acquired at symptom onset. Radiomic features were determined using a bilateral segmentation of the hippocampus. The best approach we tested is based on a Lasso regression. Using independent test data, our final six-feature model achieved a very high performance in predicting serostatus: a mean AUC = 0.950, a mean accuracy = 0.892, a mean sensitivity = 0.892, a mean specificity = 0.891, a mean PPV = 0.926, and a mean NPV = 0.872.

Radiomics-based machine learning has shown great promise in neuroradiological research, as highlighted by Wagner et al.^29^. As our study shows, machine learning can reliably predict the seropositivity of suspected AE patients. To the best of our knowledge, very few studies on machine learning diagnostics in AE patients have been published to date. Xiang et al. constructed five deep learning (DL) models based on individual or combined four MRI sequences to classify the datasets used as AE, herpes simplex virus encephalitis or healthy controls^44^. Their fusion model, which is based on multi-sequence MRI, showed a significantly higher performance in identifying AE than the performance of radiologists determined for comparison. Piao et al. developed a quantitative model integrating Radiomics and MRI-derived spatial distribution features for discriminating AE and low-grade diffuse astrocytoma^43^. One of their models achieved lower performance than a senior neuroradiologist, but higher performance than a junior neuroradiologist in their test and independent validation sets. Recently, Stake et al. have shown that serostatus in AE patients can be determined based on radiomic features obtained from segmentation of the amygdala^45^. As in our current study, Radiomics-based machine learning was also used in this study. In a comparison of these two studies, segmentation of the hippocampus even seems to provide slightly better results than those obtained from segmentation of the amygdala.

The integration of AI into medical practice, particularly in neuroradiology, is a growing trend, as Lui et al.^46^ emphasize. The potential applications of Radiomics and machine learning in healthcare, particularly in medical diagnostics, appear to be limitless. For example, Coroller et al.^47^ have already shown that Radiomics can improve the detection of distant metastases in lung adenocarcinomas and significantly enhance quantitative image analysis, as described by Lambin et al.^48^. Machine learning can also shorten the time to diagnosis, favourably influencing patient morbidity and mortality. This is crucial for patients in intensive care units, as recently demonstrated by Harutyunyan et al.^49^. Another advantage of ML-based diagnosis is its often non-invasive nature, as Chen et al. emphasize^50^. This means that more precise, personalized medicine can be defined and tailored to individual patients. However, it has also been shown that the reliable use of machine learning models still requires a high level of technical expertise for their development^51^.

Our study has several limitations that need to be considered. Firstly, its retrospective character and lack of a normal control group should be mentioned. Secondly, due to insufficient image quality (e.g. artifacts) and the fact that some of the MRI images originated from external centers with different standards of MRI diagnostics, a total of 11 patients had to be excluded from our analyses. Although our study cohort of 98 patients is comparatively large for a study on autoimmune encephalitis, it is relatively small for developing machine learning models. We therefore used a technique with 100 runs and were able to show that our model results are very stable in terms of both feature composition and performance. In addition, the number of features available for model development was significantly reduced by using a correlation filter and the number of final model features was minimized using the techniques described in the manuscript. These are important techniques to reduce the likelihood of overfitting. Nevertheless, larger prospective studies are essential to validate our results and to achieve even more reliable model predictions. Third, the bilateral segmentation of the hippocampus was performed manually, which may be associated with a risk of inherent bias. As Heine et al.^27^ stated, many image morphological abnormalities of AE occur in the hippocampus, prompting us to restrict our study to this anatomical structure. Finally, due to the possibility of false-positive antibody test results, the possible overestimation of the clinical significance of seropositivity must be taken into account. Conversely, due to the approach we used to screen our patient database, an overinterpretation of our seronegative patient cohort cannot be completely ruled out either. High heterogeneity of radiomics input data also needs to be considered. Furthermore, there are several hurdles (e.g. need for manual segmentation) that severely limit clinical implementation of our radiomics approach. Despite these limitations, our Radiomics-based machine learning models provided very promising results in predicting the serostatus of patients with suspected AE using unknown test data.

## Conclusion

Our proof-of-concept study demonstrates that Radiomics-based machine learning can reliably predict serostatus in suspected autoimmune encephalitis (AE). Such methods could potentially help to optimize the allocation of laboratory resources by targeting antibody diagnostics to seropositive cases in the future. However, further prospective studies with significantly larger cohorts are initially required to validate and confirm our results.

## Data Availability

The datasets used and/or analysed during the current study are available from the corresponding author on reasonable request.
